# Supplementary material for: From Feeding Challenges to Oral-Motor Dyspraxia: A Comprehensive Description of 10 New Cases with CTNNB1 Syndrome
Source: Genes (Basel). 2023 Sep 22;14(10):1843. doi: 10.3390/genes14101843 (PMC10606760; doi:10.3390/genes14101843)
Supplement: Supplementary file 1 [file genes-14-01843-s001.zip › genes-2593491-supplementary.pdf]

Supplementary Table 1. Anthropometric findings in our cohort of 10 cases with *CTNNB1* syndrome.

		WEIGHT		HIGHT		BMI		CC
**PT**	**Age (y)**	**value (kg)**	**%ile**	**value (cm)**	%ile	value	%ile	value (cm), SD
**1**	6	16.3	6%	102.0	2%	15.1	47%	46.0, **-3.7**
**2**	6	24.9	87%	121.0	84%	17.0	**85%**	49.0, -1.8
**3**	23	55.2	4%	167.5	10%	19.7	9%	53.5, -1.9
**4**	15.5	47.0	10%	170.5	45%	16.2	**2%**	49.5, **-3.6**
**5**	4.5	13.0	1%	99.0	8%	13.3	**1%**	45.6, **-3.2**
**6**	5.5	19.0	42%	107.3	10%	16.5	81%	49.0, -1.8
**7**	10.5	35.7	57%	138.7	32%	18.6	74%	49.5, **-2.7**
**8**	10	22.0	0%	121.6	0%	14.9	13%	46.0, **-5.3**
**9**	9	34.3	94%	135.5	90%	18.7	**90%**	49.5, **-2.4**
**10**	10.5	28.1	11%	138.5	37%	14.6	9%	49.0, **-2.5**

BMI= body mass index; CC= head cephalic circumference; Pt=patients; SD= standard deviation; Y=years. In bold: pathological score
